# 1-{6-Chloro-2-[(2-chloro-6-methyl­quinolin-3-yl)meth­oxy]-4-phenyl­quinolin-3-yl}ethanone

**DOI:** 10.1107/S1600536810022701

**Published:** 2010-06-18

**Authors:** F. Nawaz Khan, Venkatesha R. Hathwar, Rajesh Kumar, Atul Kumar Kushwaha, Mehmet Akkurt

**Affiliations:** a Organic and Medicinal Chemistry Research Laboratory, Organic Chemistry Division, School of Advanced Sciences, VIT University, Vellore 632 014, Tamil Nadu, India; b Solid State and Structural Chemistry Unit, Indian Institute of Science, Bangalore 560 012, Karnataka, India; c Department of Physics, Faculty of Arts and Sciences, Erciyes University, 38039 Kayseri, Turkey

## Abstract

In the title compound, C_28_H_20_Cl_2_N_2_O_2_, the 2-chloro­quinoline and 6-chloro­quinoline ring systems are twisted slightly, making a dihedral angle of 4.05 (3)°. The dihedral angle between the 2-quinoline ring system and the phenyl ring attached to it is 74.43 (5)°. In the crystal structure, a pair of inter­molecular C—H⋯O hydrogen bonds connect the mol­ecules, forming centrosymmetric dimers with *R*_2_^2^(16) motifs. The dimers are further consolidated by a C—H⋯π inter­action and a π–π stacking inter­action with a centroid–centroid distance of 3.6562 (10) Å.

## Related literature

For related structures, see: Khan, Roopan, Hathwar & Akkurt (2010 ▶); Khan, Roopan, Kumar *et al.* (2010 ▶); Roopan & Khan (2009 ▶). For the biological acivity of 2-quinolone derivatives, see: Ukita & Mizuno (1960 ▶); Jayashree *et al.* (2010 ▶); Joseph *et al.* (2002 ▶); Xiao *et al.* (2001 ▶). For hydrogen-bond motifs, see: Bernstein *et al.* (1995 ▶).
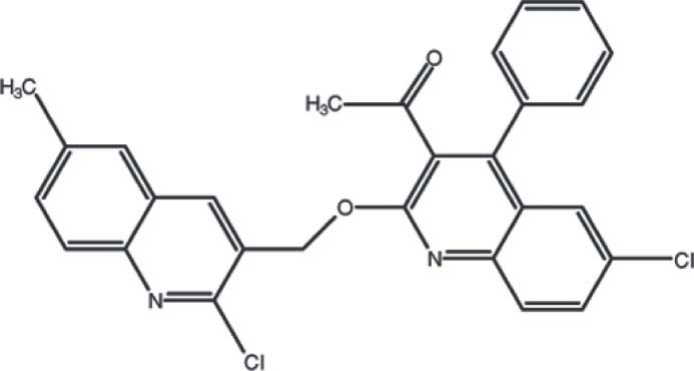


## Experimental

### 

#### Crystal data

C_28_H_20_Cl_2_N_2_O_2_
*M* *_r_* = 487.36
Triclinic, 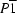

*a* = 8.0552 (2) Å
*b* = 12.4499 (5) Å
*c* = 13.3718 (5) Å
α = 67.555 (4)°
β = 80.183 (3)°
γ = 77.273 (3)°
*V* = 1203.40 (8) Å^3^
*Z* = 2
Mo *K*α radiation
μ = 0.30 mm^−1^
*T* = 295 K
0.24 × 0.18 × 0.17 mm

#### Data collection

Oxford Xcalibur Eos (Nova) CCD detector diffractometer
Absorption correction: multi-scan (*CrysAlis PRO RED*; Oxford Diffraction, 2009 ▶) *T*_min_ = 0.912, *T*_max_ = 0.951
23521 measured reflections
4468 independent reflections
3013 reflections with *I* > 2σ(*I*)
*R*_int_ = 0.034

#### Refinement

*R*[*F* ^2^ > 2σ(*F* ^2^)] = 0.038
*wR*(*F* ^2^) = 0.103
*S* = 1.05
4468 reflections
309 parameters
H-atom parameters constrained
Δρ_max_ = 0.18 e Å^−3^
Δρ_min_ = −0.20 e Å^−3^

### 

Data collection: *CrysAlis PRO CCD* (Oxford Diffraction, 2009 ▶); cell refinement: *CrysAlis PRO CCD*; data reduction: *CrysAlis PRO RED* (Oxford Diffraction, 2009 ▶); program(s) used to solve structure: *SHELXS97* (Sheldrick, 2008 ▶); program(s) used to refine structure: *SHELXL97* (Sheldrick, 2008 ▶); molecular graphics: *ORTEP-3* (Farrugia, 1997 ▶); software used to prepare material for publication: *WinGX* (Farrugia, 1999 ▶), *PARST* (Nardelli, 1983 ▶) and *PLATON* (Spek, 2009 ▶).

## Supplementary Material

Crystal structure: contains datablocks global, I. DOI: 10.1107/S1600536810022701/is2563sup1.cif

Structure factors: contains datablocks I. DOI: 10.1107/S1600536810022701/is2563Isup2.hkl

Additional supplementary materials:  crystallographic information; 3D view; checkCIF report

## Figures and Tables

**Table 1:** Hydrogen-bond geometry (Å, °) *Cg*4 is the centroid of the C14–C19 ring.

*D*—H⋯*A*	*D*—H	H⋯*A*	*D*⋯*A*	*D*—H⋯*A*
C3—H3⋯O1	0.93	2.39	2.735 (2)	101
C24—H24⋯O2^i^	0.93	2.51	3.392 (3)	157
C10—H10*A*⋯*Cg*4^ii^	0.97	2.67	3.4430 (18)	137

Symmetry codes: (i) 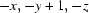; (ii) 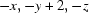.

**Fig. 1.**
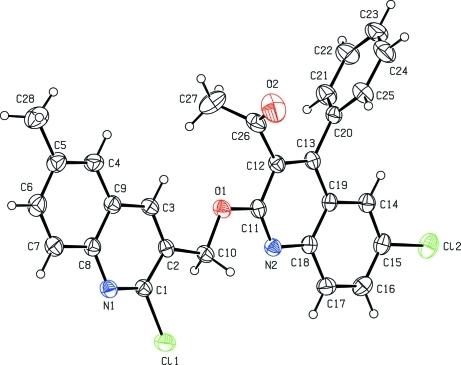
A general view of the title compound, showing the atom-numbering scheme. Displacement ellipsoids are drawn at the 30% probability level.

**Fig. 2.**
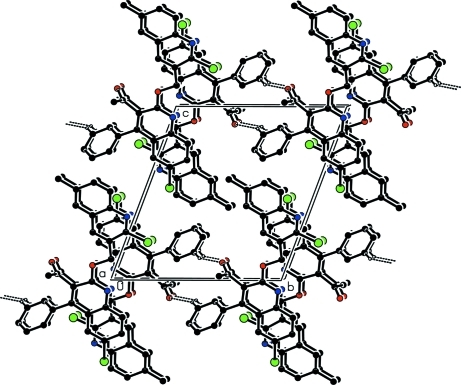
Molecular packing of the title compound with hydrogen bonding shown as dashed lines.

## supplementary crystallographic information

### Comment

In continuation of our previous work (Roopan *et al.*, 2009; Khan, Roopan, Hathwar & Akkurt, 2010; Khan, Roopan, Kumar *et al.*, 2010), we here report the crystal and molecular structures of 1-{2-[(2-chloro-6-methylquinolin-3-yl)methoxy]-6-chloro-4-phenylquinolin-3-yl}ethanone, (I).

In the title molecule, geometric parameters are in the usual ranges (Fig. 1). The 2-chloroquinoline (N1/C1–C9/Cl1) and 6-chloroquinoline (N2/C11—C19/Cl2) rings are approximately planar, with maximal deviations from their mean planes of -0.070 (1) and of -0.027 (1) Å for Cl1 and Cl2 atoms, respectively. The dihedral angle between these rings is 4.05 (3)°. The N2/C11—C19 quinoline ring makes dihedral angles of 74.43 (5) and 83.79 (11)° with the C20–C25 phenyl ring and the C26/C27/O2 acetaldehyde group, respectively.

In the crystal structure, intermolecular C—H···O hydrogen bonds link the pairs of molecules through an inversion center, forming a pseudo-dimer with an *R*^2^_2_(16) (Table 1 and Fig. 2; Bernstein *et al.*, 1995). C—H···π interactions (Table 1) and π–π stacking interactions between the quinoline rings [*Cg*1···*Cg*4(1 - *x*, 2 - *y*, -*z*) = 3.6562 (10) Å; *Cg*1 and *Cg*4 are the centroids of the N1/C1–C3/C8/C9 pyridine and C14–C19 benzene rings, respectively] may further stabilize the structure.

### Experimental

To a solution of 1-(6-chloro-2-hydroxy-4-phenylquinolin-3-yl)ethanone (1 mmol) in DMSO (5 ml), 2-chloro-3-chloromethyl-6-methylquinoline (1 mmol) and Ag_2_SO_4_ (10 mol %) were added and refluxed at 383 K. The reaction was completed with in 20 min. The reaction mixture was then filtered and the supernatant liquid was added drop wise in to the crushed ice. The solution was neutralized with dilute HCl. The precipitate was filtered off and re-crystallized with ethanol. The clear solution was kept for a day and the resulting crystals were dried.

### Refinement

All H atoms were positioned with idealized geometry (C—H = 0.93–0.97 Å) and were refined as riding, with *U*_iso_(H) = 1.2–1.5*U*_eq_(C).

### Crystal data

**Table:**

C_28_H_20_Cl_2_N_2_O_2_	*Z* = 2
*M**_r_* = 487.36	*F*(000) = 504
Triclinic, *P*1	*D*_x_ = 1.345 Mg m^−^^3^
Hall symbol: -P 1	Mo *K*α radiation, λ = 0.71073 Å
*a* = 8.0552 (2) Å	Cell parameters from 1523 reflections
*b* = 12.4499 (5) Å	θ = 1.9–21.4°
*c* = 13.3718 (5) Å	µ = 0.30 mm^−^^1^
α = 67.555 (4)°	*T* = 295 K
β = 80.183 (3)°	Block, colourless
γ = 77.273 (3)°	0.24 × 0.18 × 0.17 mm
*V* = 1203.40 (8) Å^3^	

### Data collection

**Table:**

Oxford Xcalibur Eos (Nova) CCD detector diffractometer	4468 independent reflections
Radiation source: Enhance (Mo) X-ray Source	3013 reflections with *I* > 2σ(*I*)
graphite	*R*_int_ = 0.034
ω scans	θ_max_ = 25.5°, θ_min_ = 3.2°
Absorption correction: multi-scan (*CrysAlis PRO RED*; Oxford Diffraction, 2009)	*h* = −9→9
*T*_min_ = 0.912, *T*_max_ = 0.951	*k* = −15→15
23521 measured reflections	*l* = −16→16

### Refinement

**Table:**

Refinement on *F*^2^	Primary atom site location: structure-invariant direct methods
Least-squares matrix: full	Secondary atom site location: difference Fourier map
*R*[*F*^2^ > 2σ(*F*^2^)] = 0.038	Hydrogen site location: inferred from neighbouring sites
*wR*(*F*^2^) = 0.103	H-atom parameters constrained
*S* = 1.05	*w* = 1/[σ^2^(*F*_o_^2^) + (0.052*P*)^2^] where *P* = (*F*_o_^2^ + 2*F*_c_^2^)/3
4468 reflections	(Δ/σ)_max_ = 0.001
309 parameters	Δρ_max_ = 0.18 e Å^−^^3^
0 restraints	Δρ_min_ = −0.20 e Å^−^^3^

### Special details

**Table:**

Geometry. All e.s.d.'s (except the e.s.d. in the dihedral angle between two l.s. planes) are estimated using the full covariance matrix. The cell e.s.d.'s are taken into account individually in the estimation of e.s.d.'s in distances, angles and torsion angles; correlations between e.s.d.'s in cell parameters are only used when they are defined by crystal symmetry. An approximate (isotropic) treatment of cell e.s.d.'s is used for estimating e.s.d.'s involving l.s. planes.
Refinement. Refinement of *F*^2^ against ALL reflections. The weighted *R*-factor *wR* and goodness of fit *S* are based on *F*^2^, conventional *R*-factors *R* are based on *F*, with *F* set to zero for negative *F*^2^. The threshold expression of *F*^2^ > σ(*F*^2^) is used only for calculating *R*-factors(gt) *etc*. and is not relevant to the choice of reflections for refinement. *R*-factors based on *F*^2^ are statistically about twice as large as those based on *F*, and *R*- factors based on ALL data will be even larger.

### Fractional atomic coordinates and isotropic or equivalent isotropic displacement parameters (Å^2^)

**Table:**

	*x*	*y*	*z*	*U*_iso_*/*U*_eq_	
Cl1	0.33663 (7)	1.14088 (4)	0.22132 (4)	0.06059 (17)	
Cl2	−0.09000 (6)	1.14116 (5)	−0.47007 (4)	0.06921 (19)	
N2	0.21161 (17)	1.00876 (13)	−0.06019 (11)	0.0423 (4)	
O1	0.33714 (14)	0.86813 (10)	0.08785 (9)	0.0475 (3)	
N1	0.49540 (17)	0.96473 (13)	0.36950 (11)	0.0448 (4)	
C19	0.13345 (19)	0.94637 (15)	−0.19597 (13)	0.0385 (4)	
C14	0.0612 (2)	0.98122 (16)	−0.29553 (13)	0.0444 (4)	
H14	0.0544	0.9251	−0.3241	0.053*	
C2	0.4134 (2)	0.91455 (15)	0.22883 (13)	0.0400 (4)	
C9	0.5542 (2)	0.76123 (16)	0.37802 (14)	0.0439 (4)	
C8	0.5616 (2)	0.84815 (16)	0.42010 (14)	0.0437 (4)	
C3	0.4784 (2)	0.79952 (16)	0.27944 (14)	0.0446 (4)	
H3	0.4730	0.7447	0.2489	0.054*	
C18	0.1429 (2)	1.03427 (15)	−0.15555 (13)	0.0403 (4)	
C13	0.1931 (2)	0.82572 (15)	−0.13202 (13)	0.0391 (4)	
C1	0.4259 (2)	0.99192 (15)	0.28090 (14)	0.0416 (4)	
C10	0.3332 (2)	0.96331 (15)	0.12404 (13)	0.0430 (4)	
H10A	0.2160	1.0009	0.1346	0.052*	
H10B	0.3956	1.0218	0.0700	0.052*	
C12	0.2581 (2)	0.80213 (15)	−0.03618 (13)	0.0412 (4)	
C7	0.6397 (2)	0.81471 (18)	0.51636 (14)	0.0537 (5)	
H7	0.6455	0.8713	0.5446	0.064*	
C11	0.2656 (2)	0.89904 (16)	−0.00647 (13)	0.0407 (4)	
C15	0.0018 (2)	1.09715 (17)	−0.34893 (13)	0.0487 (5)	
C17	0.0794 (2)	1.15241 (16)	−0.21340 (15)	0.0521 (5)	
H17	0.0848	1.2103	−0.1867	0.062*	
C16	0.0096 (2)	1.18333 (17)	−0.30877 (15)	0.0565 (5)	
H16	−0.0327	1.2620	−0.3467	0.068*	
C6	0.7064 (2)	0.70026 (18)	0.56792 (15)	0.0588 (5)	
H6	0.7580	0.6797	0.6313	0.071*	
C20	0.1809 (2)	0.72977 (15)	−0.17002 (13)	0.0418 (4)	
C26	0.3230 (3)	0.67904 (17)	0.03888 (15)	0.0570 (5)	
C4	0.6242 (2)	0.64294 (17)	0.43520 (15)	0.0542 (5)	
H4	0.6185	0.5850	0.4086	0.065*	
C5	0.7000 (2)	0.61141 (18)	0.52858 (15)	0.0571 (5)	
C25	0.0585 (3)	0.65958 (17)	−0.12159 (15)	0.0589 (5)	
H25	−0.0141	0.6695	−0.0626	0.071*	
C21	0.2876 (2)	0.71344 (18)	−0.25780 (15)	0.0588 (5)	
H21	0.3704	0.7608	−0.2918	0.071*	
O2	0.2265 (2)	0.62082 (15)	0.10414 (13)	0.0992 (6)	
C22	0.2719 (3)	0.6277 (2)	−0.29494 (18)	0.0738 (6)	
H22	0.3451	0.6169	−0.3534	0.089*	
C23	0.1503 (3)	0.5586 (2)	−0.2469 (2)	0.0773 (7)	
H23	0.1401	0.5010	−0.2727	0.093*	
C24	0.0425 (3)	0.57386 (19)	−0.16021 (19)	0.0754 (7)	
H24	−0.0411	0.5268	−0.1275	0.090*	
C27	0.5116 (3)	0.6362 (2)	0.0300 (2)	0.0923 (8)	
H27A	0.5383	0.5606	0.0860	0.138*	
H27B	0.5703	0.6913	0.0381	0.138*	
H27C	0.5474	0.6293	−0.0399	0.138*	
C28	0.7750 (3)	0.48492 (19)	0.58949 (19)	0.0845 (7)	
H28A	0.7624	0.4363	0.5512	0.127*	
H28B	0.7162	0.4588	0.6611	0.127*	
H28C	0.8941	0.4793	0.5950	0.127*	

### Atomic displacement parameters (Å^2^)

**Table:**

	*U*^11^	*U*^22^	*U*^33^	*U*^12^	*U*^13^	*U*^23^
Cl1	0.0836 (4)	0.0442 (3)	0.0574 (3)	−0.0070 (2)	−0.0237 (3)	−0.0171 (2)
Cl2	0.0722 (4)	0.0812 (4)	0.0445 (3)	−0.0019 (3)	−0.0246 (2)	−0.0100 (3)
N2	0.0451 (8)	0.0450 (10)	0.0386 (8)	−0.0081 (7)	−0.0068 (7)	−0.0157 (7)
O1	0.0620 (8)	0.0483 (8)	0.0390 (7)	−0.0093 (6)	−0.0166 (6)	−0.0181 (6)
N1	0.0521 (9)	0.0484 (10)	0.0398 (8)	−0.0080 (7)	−0.0091 (7)	−0.0205 (8)
C19	0.0351 (9)	0.0472 (11)	0.0334 (9)	−0.0073 (8)	−0.0036 (7)	−0.0144 (8)
C14	0.0439 (10)	0.0534 (12)	0.0373 (10)	−0.0080 (9)	−0.0052 (8)	−0.0175 (9)
C2	0.0401 (9)	0.0486 (11)	0.0359 (9)	−0.0117 (8)	−0.0032 (8)	−0.0181 (9)
C9	0.0456 (10)	0.0503 (12)	0.0403 (10)	−0.0063 (9)	−0.0064 (8)	−0.0211 (9)
C8	0.0440 (10)	0.0524 (12)	0.0388 (10)	−0.0068 (9)	−0.0052 (8)	−0.0212 (9)
C3	0.0507 (10)	0.0484 (12)	0.0436 (10)	−0.0077 (9)	−0.0069 (8)	−0.0255 (9)
C18	0.0377 (9)	0.0459 (11)	0.0373 (10)	−0.0078 (8)	−0.0043 (8)	−0.0140 (9)
C13	0.0386 (9)	0.0460 (11)	0.0347 (9)	−0.0119 (8)	−0.0030 (7)	−0.0144 (8)
C1	0.0444 (10)	0.0426 (10)	0.0401 (10)	−0.0088 (8)	−0.0049 (8)	−0.0160 (8)
C10	0.0498 (10)	0.0458 (11)	0.0398 (10)	−0.0109 (8)	−0.0059 (8)	−0.0200 (9)
C12	0.0463 (10)	0.0434 (11)	0.0353 (9)	−0.0123 (8)	−0.0066 (8)	−0.0119 (8)
C7	0.0605 (12)	0.0627 (14)	0.0458 (11)	−0.0061 (10)	−0.0136 (9)	−0.0270 (10)
C11	0.0410 (10)	0.0496 (12)	0.0334 (9)	−0.0115 (8)	−0.0052 (8)	−0.0143 (9)
C15	0.0459 (10)	0.0596 (13)	0.0349 (10)	−0.0052 (9)	−0.0093 (8)	−0.0105 (9)
C17	0.0589 (12)	0.0437 (11)	0.0539 (12)	−0.0052 (9)	−0.0116 (10)	−0.0171 (10)
C16	0.0598 (12)	0.0473 (12)	0.0517 (12)	−0.0009 (9)	−0.0127 (10)	−0.0074 (10)
C6	0.0615 (12)	0.0701 (15)	0.0443 (11)	−0.0030 (11)	−0.0170 (9)	−0.0196 (11)
C20	0.0495 (10)	0.0418 (10)	0.0359 (9)	−0.0069 (9)	−0.0151 (8)	−0.0116 (8)
C26	0.0881 (16)	0.0481 (12)	0.0417 (11)	−0.0198 (12)	−0.0261 (11)	−0.0109 (10)
C4	0.0634 (12)	0.0479 (12)	0.0554 (12)	−0.0011 (10)	−0.0138 (10)	−0.0241 (10)
C5	0.0597 (12)	0.0544 (13)	0.0519 (12)	−0.0025 (10)	−0.0130 (10)	−0.0139 (10)
C25	0.0715 (13)	0.0595 (13)	0.0526 (12)	−0.0254 (11)	−0.0058 (10)	−0.0200 (11)
C21	0.0648 (12)	0.0685 (14)	0.0541 (12)	−0.0164 (11)	−0.0020 (10)	−0.0328 (11)
O2	0.1195 (14)	0.0826 (12)	0.0773 (11)	−0.0532 (11)	−0.0305 (10)	0.0200 (10)
C22	0.0899 (16)	0.0798 (17)	0.0691 (15)	−0.0104 (14)	−0.0101 (13)	−0.0470 (14)
C23	0.1044 (18)	0.0630 (15)	0.0848 (17)	−0.0105 (14)	−0.0340 (15)	−0.0405 (14)
C24	0.0909 (16)	0.0651 (16)	0.0809 (17)	−0.0361 (13)	−0.0186 (14)	−0.0211 (13)
C27	0.0977 (19)	0.0612 (15)	0.0880 (18)	0.0178 (13)	−0.0256 (14)	−0.0039 (13)
C28	0.1036 (18)	0.0600 (15)	0.0795 (16)	0.0047 (13)	−0.0368 (14)	−0.0123 (13)

### Geometric parameters (Å, °)

**Table:**

Cl1—C1	1.7523 (18)	C7—H7	0.9300
Cl2—C15	1.7388 (17)	C15—C16	1.387 (3)
N2—C11	1.291 (2)	C17—C16	1.366 (2)
N2—C18	1.374 (2)	C17—H17	0.9300
O1—C11	1.3603 (19)	C16—H16	0.9300
O1—C10	1.4336 (19)	C6—C5	1.408 (3)
N1—C1	1.292 (2)	C6—H6	0.9300
N1—C8	1.372 (2)	C20—C25	1.373 (2)
C19—C18	1.413 (2)	C20—C21	1.385 (2)
C19—C14	1.418 (2)	C26—O2	1.194 (2)
C19—C13	1.435 (2)	C26—C27	1.495 (3)
C14—C15	1.359 (2)	C4—C5	1.366 (2)
C14—H14	0.9300	C4—H4	0.9300
C2—C3	1.354 (2)	C5—C28	1.509 (3)
C2—C1	1.415 (2)	C25—C24	1.388 (3)
C2—C10	1.495 (2)	C25—H25	0.9300
C9—C4	1.412 (2)	C21—C22	1.374 (3)
C9—C8	1.413 (2)	C21—H21	0.9300
C9—C3	1.416 (2)	C22—C23	1.359 (3)
C8—C7	1.406 (2)	C22—H22	0.9300
C3—H3	0.9300	C23—C24	1.373 (3)
C18—C17	1.399 (2)	C23—H23	0.9300
C13—C12	1.370 (2)	C24—H24	0.9300
C13—C20	1.492 (2)	C27—H27A	0.9600
C10—H10A	0.9700	C27—H27B	0.9600
C10—H10B	0.9700	C27—H27C	0.9600
C12—C11	1.422 (2)	C28—H28A	0.9600
C12—C26	1.510 (2)	C28—H28B	0.9600
C7—C6	1.354 (3)	C28—H28C	0.9600
			
C11—N2—C18	116.95 (14)	C16—C17—C18	120.51 (17)
C11—O1—C10	115.17 (13)	C16—C17—H17	119.7
C1—N1—C8	116.89 (14)	C18—C17—H17	119.7
C18—C19—C14	118.60 (16)	C17—C16—C15	119.85 (18)
C18—C19—C13	117.98 (14)	C17—C16—H16	120.1
C14—C19—C13	123.39 (15)	C15—C16—H16	120.1
C15—C14—C19	119.59 (16)	C7—C6—C5	122.07 (18)
C15—C14—H14	120.2	C7—C6—H6	119.0
C19—C14—H14	120.2	C5—C6—H6	119.0
C3—C2—C1	115.83 (15)	C25—C20—C21	118.83 (17)
C3—C2—C10	124.90 (15)	C25—C20—C13	120.46 (15)
C1—C2—C10	119.26 (15)	C21—C20—C13	120.64 (16)
C4—C9—C8	118.56 (16)	O2—C26—C27	122.3 (2)
C4—C9—C3	124.18 (16)	O2—C26—C12	120.49 (19)
C8—C9—C3	117.25 (16)	C27—C26—C12	117.21 (18)
N1—C8—C7	118.82 (16)	C5—C4—C9	121.69 (17)
N1—C8—C9	121.91 (15)	C5—C4—H4	119.2
C7—C8—C9	119.27 (17)	C9—C4—H4	119.2
C2—C3—C9	121.12 (16)	C4—C5—C6	118.33 (18)
C2—C3—H3	119.4	C4—C5—C28	121.61 (19)
C9—C3—H3	119.4	C6—C5—C28	120.06 (18)
N2—C18—C17	117.78 (15)	C20—C25—C24	120.35 (19)
N2—C18—C19	122.61 (15)	C20—C25—H25	119.8
C17—C18—C19	119.61 (15)	C24—C25—H25	119.8
C12—C13—C19	118.40 (15)	C22—C21—C20	120.46 (19)
C12—C13—C20	121.80 (15)	C22—C21—H21	119.8
C19—C13—C20	119.80 (14)	C20—C21—H21	119.8
N1—C1—C2	126.97 (16)	C23—C22—C21	120.5 (2)
N1—C1—Cl1	115.41 (13)	C23—C22—H22	119.8
C2—C1—Cl1	117.62 (13)	C21—C22—H22	119.8
O1—C10—C2	108.45 (14)	C22—C23—C24	120.0 (2)
O1—C10—H10A	110.0	C22—C23—H23	120.0
C2—C10—H10A	110.0	C24—C23—H23	120.0
O1—C10—H10B	110.0	C23—C24—C25	119.9 (2)
C2—C10—H10B	110.0	C23—C24—H24	120.1
H10A—C10—H10B	108.4	C25—C24—H24	120.1
C13—C12—C11	118.04 (16)	C26—C27—H27A	109.5
C13—C12—C26	123.48 (15)	C26—C27—H27B	109.5
C11—C12—C26	118.48 (15)	H27A—C27—H27B	109.5
C6—C7—C8	120.07 (18)	C26—C27—H27C	109.5
C6—C7—H7	120.0	H27A—C27—H27C	109.5
C8—C7—H7	120.0	H27B—C27—H27C	109.5
N2—C11—O1	119.80 (15)	C5—C28—H28A	109.5
N2—C11—C12	125.97 (15)	C5—C28—H28B	109.5
O1—C11—C12	114.24 (15)	H28A—C28—H28B	109.5
C14—C15—C16	121.83 (17)	C5—C28—H28C	109.5
C14—C15—Cl2	120.19 (15)	H28A—C28—H28C	109.5
C16—C15—Cl2	117.97 (15)	H28B—C28—H28C	109.5
			
C18—C19—C14—C15	−0.5 (2)	C18—N2—C11—C12	−1.0 (2)
C13—C19—C14—C15	177.48 (14)	C10—O1—C11—N2	4.9 (2)
C1—N1—C8—C7	179.53 (15)	C10—O1—C11—C12	−175.04 (13)
C1—N1—C8—C9	−0.2 (2)	C13—C12—C11—N2	2.2 (3)
C4—C9—C8—N1	−179.55 (15)	C26—C12—C11—N2	−178.15 (16)
C3—C9—C8—N1	1.3 (2)	C13—C12—C11—O1	−177.87 (13)
C4—C9—C8—C7	0.7 (2)	C26—C12—C11—O1	1.7 (2)
C3—C9—C8—C7	−178.42 (15)	C19—C14—C15—C16	−0.1 (3)
C1—C2—C3—C9	−0.3 (2)	C19—C14—C15—Cl2	−179.06 (11)
C10—C2—C3—C9	179.17 (15)	N2—C18—C17—C16	179.97 (15)
C4—C9—C3—C2	179.92 (16)	C19—C18—C17—C16	−0.4 (2)
C8—C9—C3—C2	−1.0 (2)	C18—C17—C16—C15	−0.2 (3)
C11—N2—C18—C17	178.29 (14)	C14—C15—C16—C17	0.4 (3)
C11—N2—C18—C19	−1.3 (2)	Cl2—C15—C16—C17	179.46 (13)
C14—C19—C18—N2	−179.64 (14)	C8—C7—C6—C5	−0.3 (3)
C13—C19—C18—N2	2.2 (2)	C12—C13—C20—C25	74.1 (2)
C14—C19—C18—C17	0.8 (2)	C19—C13—C20—C25	−105.04 (19)
C13—C19—C18—C17	−177.35 (14)	C12—C13—C20—C21	−108.88 (19)
C18—C19—C13—C12	−0.9 (2)	C19—C13—C20—C21	72.0 (2)
C14—C19—C13—C12	−178.94 (14)	C13—C12—C26—O2	−84.5 (2)
C18—C19—C13—C20	178.25 (14)	C11—C12—C26—O2	95.9 (2)
C14—C19—C13—C20	0.2 (2)	C13—C12—C26—C27	97.5 (2)
C8—N1—C1—C2	−1.4 (3)	C11—C12—C26—C27	−82.1 (2)
C8—N1—C1—Cl1	177.98 (11)	C8—C9—C4—C5	−0.9 (3)
C3—C2—C1—N1	1.6 (3)	C3—C9—C4—C5	178.22 (16)
C10—C2—C1—N1	−177.90 (15)	C9—C4—C5—C6	0.4 (3)
C3—C2—C1—Cl1	−177.69 (12)	C9—C4—C5—C28	−179.89 (17)
C10—C2—C1—Cl1	2.8 (2)	C7—C6—C5—C4	0.2 (3)
C11—O1—C10—C2	179.04 (12)	C7—C6—C5—C28	−179.53 (19)
C3—C2—C10—O1	0.1 (2)	C21—C20—C25—C24	0.0 (3)
C1—C2—C10—O1	179.58 (13)	C13—C20—C25—C24	177.12 (18)
C19—C13—C12—C11	−1.1 (2)	C25—C20—C21—C22	−0.6 (3)
C20—C13—C12—C11	179.73 (14)	C13—C20—C21—C22	−177.68 (18)
C19—C13—C12—C26	179.30 (15)	C20—C21—C22—C23	0.8 (3)
C20—C13—C12—C26	0.1 (3)	C21—C22—C23—C24	−0.3 (4)
N1—C8—C7—C6	−179.90 (15)	C22—C23—C24—C25	−0.2 (4)
C9—C8—C7—C6	−0.2 (3)	C20—C25—C24—C23	0.4 (3)
C18—N2—C11—O1	179.12 (13)		

### Hydrogen-bond geometry (Å, °)

**Table:**

Cg4 is the centroid of the C14–C19 ring.

**Table:**

*D*—H···*A*	*D*—H	H···*A*	*D*···*A*	*D*—H···*A*
C3—H3···O1	0.93	2.39	2.735 (2)	101
C24—H24···O2^i^	0.93	2.51	3.392 (3)	157
C10—H10A···Cg4^ii^	0.97	2.67	3.4430 (18)	137

Symmetry codes: (i) −*x*, −*y*+1, −*z*; (ii) −*x*, −*y*+2, −*z*.
